# Multimodal targets of (‐)‐α‐thujone mediating avoidance behavior of a stored‐products pest, *Tribolium castaneum* , in response to irritant stimuli

**DOI:** 10.1002/ps.70995

**Published:** 2026-06-08

**Authors:** Kenji Shimomura, Yuto Suzuki, Soshi Ino, Takehito Terajima, Motohiro Tomizawa

**Affiliations:** ^1^ Department of Chemistry for Life Sciences and Agriculture, Faculty of Life Sciences Tokyo University of Agriculture Setagaya Japan

**Keywords:** avoidance behavior, multimodal targets, Orco receptor, Rdl receptor, (−)‐α‐thujone, TRP channel

## Abstract

**BACKGROUND:**

The reciprocal evolutionary processes among phytophagous insects and host plants involve host shift in the herbivorous insects in response to defense chemicals, such as plant‐derived terpenoid compounds that comprise a key element for adaptive radiation, leading to chemosensory diversification. To reveal the multimodal chemosensory targets mediating avoidance behavior of insects in response to irritant stimuli, in this study, we explored multimodal target molecules toward (‐)‐α‐thujone, a bicyclic monoterpenoid compound, focusing on the red flour beetle, *Tribolium castaneum*.

**RESULTS:**

The area‐preference test revealed significant avoidance behavior in adult *T. castaneum* toward (‐)‐α‐thujone. We applied systemic RNAi methodology to knock down hypothesized targets; knocking down the resistance to a dieldrin (Rdl) subunit comprising a γ‐aminobutyric acid (GABA)‐activated receptor significantly suppressed the avoidance behavior toward (‐)‐α‐thujone, rather than *l*‐menthol. Avoidance behaviors were significantly suppressed after knocking down thermo (*TcTRPM*) and non‐thermo (*TcTRPL*) TRP channels. Subsequently, quantitative reverse transcription (qRT)‐PCR‐based spatial expression analysis of *TcRdl* and *TcTRPL* revealed high *TcRdl* transcript expression in the head, followed by the antenna, and prominent *TcTRPL* transcript expression in the head. Furthermore, the reduced avoidance behavior in antenna‐dissected and knockdown of the olfactory receptor coreceptor (*TcOrco*) lines confirmed the involvement of antenna in the underlying mechanism.

**CONCLUSION:**

The results reflected potential molecular targets including non‐thermo TRP channel involved in (‐)‐α‐thujone‐mediated avoidance behavior in *T. castaneum* and indicated multimodality, as exhibited by *N*,*N*‐diethyl‐3‐methylbenzamide (DEET), the gold standard of repellent, providing useful insight for further research on the development of new repellents and excito‐repellents. © 2026 The Author(s). *Pest Management Science* published by John Wiley & Sons Ltd on behalf of Society of Chemical Industry.

## Graphical abstract

We identified four kinds of target molecules against (‐)‐α‐thujone associating avoidance behavior in *Tribolium castaneum*, indicating multimodality.

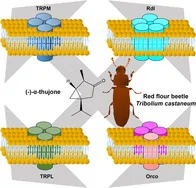

## 1 INTRODUCTION

Phytophagous insects and host plants represent diverse forms of macroevolution in the ecosystem, in which adaptions associated with defense and counter‐defense reciprocally drive evolutionary diversification.^1^ In recent decades, both have been focused on while exploring the origin of biodiversity. In reciprocal co‐evolutionary processes, host choices by herbivorous insects have been considerably shifted owing to the presence of the plant secondary metabolites involved in defense against insects, inducing coevolution with adaptive radiation.^2^ In this context, the relatively rapid radiation in host diversity among phytophagous insects potentially facilitates revealing the ecological and evolutionary mechanisms underlying multimodal chemosensory divergence.

Among defense‐related compounds, terpenoids comprise one of the most diverse groups of compounds, with ≈50 000 compounds identified to date.^3^ Various effects of terpenoid compounds on herbivores and pathogens include beneficial and lethal impacts. Many terpenoid compounds exhibit neurotoxicity and repellency, some indicating irritant stimuli on herbivorous insects, and they behaviorally respond by moving away from and avoiding these stimuli. Therefore, exploring terpenoid compounds is crucial for finding new sources of repellent and excito‐repellent; a substance that causes oriented movement away from the odor and an irritant causes movement away from the stimulus after contacting it, respectively.^4^

More than 80 years have passed since the discovery of *N,N*‐diethyl‐3‐methylbenzamide (DEET), which remains a gold standard of repellent for mosquitoes, flies, ticks and beetles. However, DEET has some drawbacks^5^; its mode‐of‐action (MoA) remains controversial, with three hypotheses formulated: (i) olfactory blockage, (ii) chemosensory detection and (iii) neurotoxicity.^6^ Such comprehensive hypotheses would be derived from a multimodal MoA involving chemosensory receptors, suggesting that multimodality is an essential qualification for the next alternative repellent and excito‐repellent.

Nociception is an ancient and evolutionarily conserved mechanism to perceive noxious stimuli such as toxic chemicals, thermal and mechanical stimuli, with consistent underlying physiological mechanisms across insects and mammals.^7^ Potentially noxious stimuli induce pain symptoms, as well as escape and avoidance behaviors in animals. Transient receptor potential (TRP) channels play a key role as nociceptors.^8^ *Tribolium castaneum*, a coleopteran beetle, perceives and showed avoidance behavior to some plant‐derived chemical compounds via TRP channels.^9^, ^10^, ^11^, ^12^ The ability to differentiate sensory modalities crucially regulates the survival of organisms in the field; however, the molecular mechanism underlying sensory multimodality, functional at the receptor levels remains unclear.

In the last two decades, insect neuroethological mechanisms underlying olfactory‐based behavioral responses have been investigated; olfactory receptor cells (ORCs) usually express one kind of odorant receptor (OR), ionotropic receptor (IR) or gustatory receptor (GR), however, some ORCs co‐express more than one OR, IR or GR, and even different types of co‐expression are found.^13^ Recent structural analysis revealed that insect ORs comprise heterocomplexes with widely expressed olfactory receptor co‐receptors (Orco), forming nonselective cation channels. Stoichiometry of OR and Orco components with a 1:3 ratio, three Orco subunits serve as scaffold content, and remaining one OR subunit bounding odor compound causes an asymmetric pore opening for cation influx.^14^ These insights into odor recognition and channel gating are suggested to contribute to the development of repellents for agricultural pest control.

**Figure 1:**
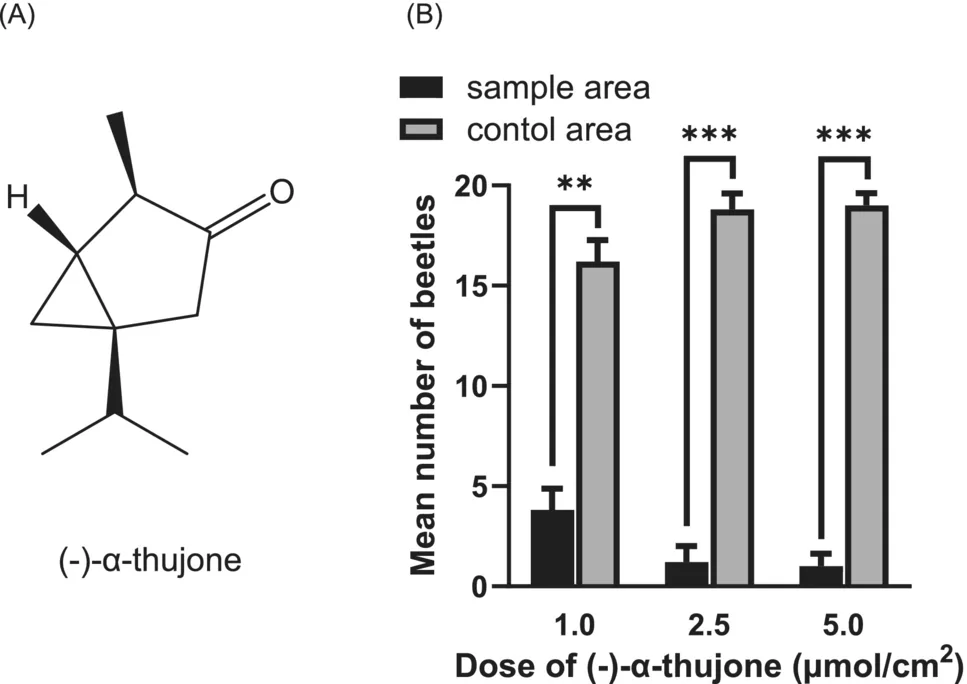
Avoidance behavior of *T. castaneum* toward (‐)‐α‐thujone. (A) Chemical structure of (‐)‐α‐thujone. (B) Twenty adults were used in the area‐preference test; data were expressed as mean ± SE of the five biological replications; asterisks above the bars indicate significant differences between the number of beetles on the (‐)‐α‐thujone‐treated area and control area (solvent applied) (paired Student's *t*‐test: **, *P* < 0.01; ***, *P* < 0.001).

Alternatively, insect γ‐aminobutyric acid (GABA)‐activated receptor comprising resistance to dieldrin (Rdl) subunit is a target for insecticides, such as fipronil and neurotoxic terpenoids.^1^ (‐)‐α‐Thujone, (1*S*,4*R*,5*R*)‐1‐isopropyl‐4‐methylbicyclo [3.1.0] hexan‐3‐one [Fig. 1(A)], is a bicyclic monoterpene compound produced by several plants, including *Salvia officinalis* (common sage), and considered a neurotoxicant that is historically derived from wormwood, connected to absinthe, a popular strong spirit aromatized with *Artemisia absinthium*, associated with convulsion, blindness, hallucination and mental deterioration.^15^ The toxicological mechanism of α‐thujone has been investigated in animals; cultured neuronal cells expressed the receptors, revealing that α‐thujone acts as an antagonist of the GABA‐gated chloride channel A type and the reversible modulator in the dorsal root ganglion.^16^ α‐thujone induced avoidance behavior in *Drosophila melanogaster*, with suggested presence of Orco‐dependent and independent mechanisms.^17^ Considering these reports, we inferred the presence of multimodal chemosensory targets of (‐)‐α‐thujone that induce avoidance behavior in insects.

In the present study, we aimed to elucidate the target molecules of (‐)‐α‐thujone, inducing avoidance behavior in *T. castaneum* by knocking down *TcRdl*, two kinds of TRP channels (*TcTRPL* and *TcTRPM*), and *TcOrco* via the systemic RNAi method.

## 2 MATERIALS AND METHODS

### 2.1 Insect

Laboratory‐maintained colonies of *T. castaneum* were used in this study. The beetles were fed on whole grain flour supplemented with 5% dried yeast in a plastic container and were maintained at 28 °C in a dark incubator.

### 2.2 Gene cloning

The whole bodies of three beetles were ground using the Biomasher II (Nippi Inc., Tokyo, Japan), followed by total RNA extraction using the ReliaPrep RNA Cell Miniprep System (Promega Corporation, Madison, WI, USA). The RNA was qualitatively and quantitatively analyzed using a NanoDrop 2000c (Thermo Fisher Scientific, Waltham, MA, USA). First‐strand cDNA was synthesized using ReverTra Ace α (TOYOBO Co., Ltd, Osaka, Japan). For the In‐Fusion cloning, full‐length *TcRdl* and *TcTrpl* coding sequences were amplified via PCR using KOD ‐Plus‐ Neo (TOYOBO) and specific primers (Supporting Information Table S1) designed based on the sequence data obtained from iBeetle‐Base.^18^, ^19^ PCR was performed on a T100 thermal cycler (Bio‐Rad, Hercules, CA, USA) using the following conditions: 94 °C for 2 min, followed by 35 cycles of 98 °C for 10 s, 58 °C for 30 s, and 68 °C for 2 min (*TcRdl*) or 4 min (*TcTrpl*). Each PCR product was separated using agarose gel electrophoresis, purified using NucleoSpin Gel and PCR Clean‐up (Takara Bio., Shiga, Japan), and assembled with a linearized pUC19 plasmid vector using the In‐Fusion Snap Assembly Master Mix (Takara Bio). The assembled DNA construct was transformed into ECOS Competent *Escherichia coli* DH5α cells (Nippon Genetics, Tokyo, Japan). *E. coli* was grown in Luria–Bertani (LB) medium; subsequently, plasmid DNA was purified using NucleoSpin Plasmid EasyPure (Takara Bio) and sequenced (Fasmac Co., Ltd, Kanagawa, Japan).

### 2.3 RNAi‐mediated gene knockdown

In order to synthesize the dsRNAs, the partial templates containing the T7 promoter sequence at the 5′ end were amplified through PCR using KOD‐Plus‐Neo with the specific primers (Table S1) under the following conditions: 94 °C for 2 min, followed by 40 cycles of 98 °C for 10 s, 58 °C for 30 s and 68 °C for 1 min. The amplicons were used as templates for dsRNA synthesis conducted using the RiboMAX Large‐Scale RNA Production System T7 (Promega). The concentration of dsRNA solutions was quantified using NanoDrop 2000c and adjusted to 2 μg μL^−1^ with RNase‐free water. *dsTcOrco* and *dsTcTrpm* were synthesized as reported previously.^9^, ^10^ dsRNA was injected into the pupa placed on the stage of a microscope using a micromanipulator (Narishige, Tokyo, Japan) equipped with a glass needle. Next, the pupae were incubated in a 24‐well plate with a piece of the aforementioned feed, at 28 °C in the dark incubator; after the adults emerged, ≈7‐day‐old adults were used in the area preference test and subsequent quantitative reverse transcription (qRT)‐PCR analysis.

### 2.4 Quantification of transcripts using qRT‐PCR

Total RNA extracted from three adults was used to confirm RNAi‐mediated knockdown. Spatial gene expression across organs was compared using specific primers (Table S1) following methods described previously.^10^ Three biological replications were performed in both experiments.

### 2.5 Area‐preference test

Avoidance behavior in adult beetles was evaluated using the area‐preference test as previously reported.^20^ (‐)‐α‐Thujone, purchased from Sigma‐Aldrich (St Louis, MO, USA), was dissolved in acetone at concentrations of 10, 25 and 50 mg mL^−1^. Filter papers (90 mm diameter) were cut in half; 500 μL sample solution were applied uniformly to one half, and 500 μL acetone were applied to the other half as a control. After the complete evaporation of the solvent, both papers were reassembled using adhesive tape and set on a 90 mm diameter glass petri dish. Subsequently, 20 beetles were released in the center of the filter paper and maintained for 3 h at 28 °C in the dark. Next, the number of beetles on each half (sample or control) of the filter paper was counted, and the avoidance index (AI) was calculated according to the following formula:

$$\text{Avoidance index}\;\left(\mathrm{AI}\right)=\left(N_{c}-N_{s}\right)/\left(N_{c}+N_{s}\right)$$

where *N*_c_ and *N*_s_ indicate the numbers of beetles present in the control and sample‐treated areas, respectively; each test included five replicates.

### 2.6 Topical application assay

Fipronil, purchased from Tokyo Chemical Industry Co., Ltd (Tokyo, Japan), was dissolved in acetone at 10 μmol mL^−1^. A topical application assay was performed using a repeating dispenser equipped with a syringe (Hamilton, Reno, NV, USA). Adult beetles treated with dsRNA were ansthetized with diethyl ether. Subsequently, 1 μL solution was topically applied to the ventral surface of the abdominal segment. After the resurgence, the beetles were set in a glass petri dish and maintained at 28 °C in a dark incubator. Mortality was recorded 3, 6, 9, 12 and 24 h post‐application. Sixty beetles were tested.

### 2.7 Statistics

The number of beetles was compared between the sample and control area using a two‐tailed paired Student's *t*‐test. The AI values and relative expression of the transcripts on each organ were compared using one‐way ANOVA, followed by the Tukey–Kramer honestly significant difference (HSD) test. The AI values of antenna‐dissected or *dsTcOrco*‐treated beetles, and relative expression of the treated transcripts were compared with the control counterpart using two‐tailed Student's or Welch's *t*‐test. These statistical analyses were performed using R software. The survival probability of beetles treated with fipronil was assessed using Kaplan–Meier survival analysis following the log‐rank (Mantel–Cox) test using prism 10 (GraphPad, Boston, MA, USA).

## 3 RESULTS

### 3.1 Avoidance behavior in response to (‐)‐α‐thujone

Initially, the area‐preference test revealed significant avoidance behaviors in *T. castaneum* in response to three (‐)‐α‐thujone at concentrations 1, 2.5 and 5 μmol cm^−2^ [Fig. 1(B)]. Based on the result, we further evaluated avoidance behavior in response to 2.5 μmol cm^−2^ (‐)‐α‐thujone using RNAi gene knockdown beetles. At these concentrations, all beetles in the petri dish were alive after 24 h.

### 3.2 Effects of *TcRdl* knocking down

**Figure 2:**
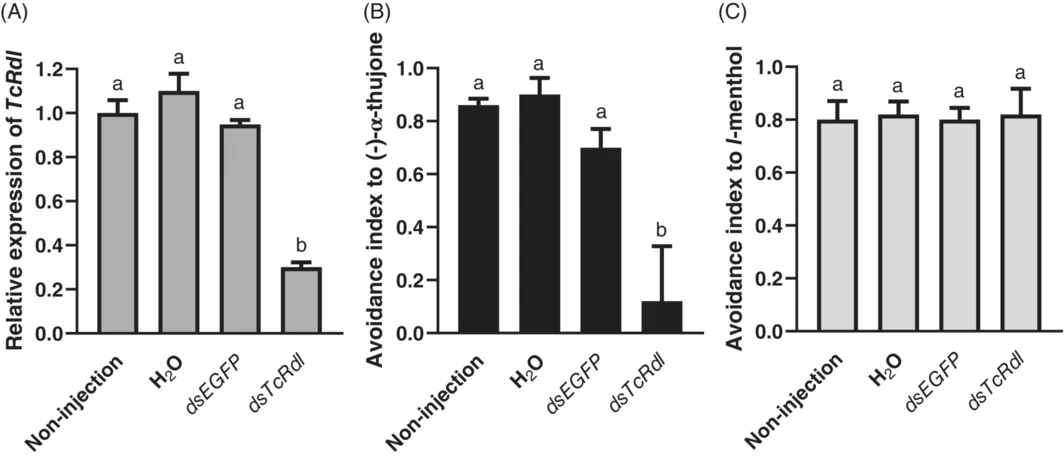
RNAi‐mediated *TcRdl* knockdown exhibited suppressed avoidance behavior in response to (‐)‐α‐thujone, a bicyclic monoterpenoid compound, but not towards *l*‐menthol, a monocyclic monoterpenoid compound. (A) *dsTcRdl* was injected into the pupae; after the adult emerged, the *TcRdl* transcripts were measured through qRT‐PCR, and the expression level was revealed as fold‐change relative to the noninjected control counterparts (n = 3). The mean avoidance indices toward (‐)‐α‐thujone (B) and *l*‐menthol (C) were compared using the area‐preference test. Data are expressed as mean ± SEM (n = 5). The same letters above the bars indicate no significant difference at *P* > 0.05 (one‐way ANOVA and Tukey–Kramer HSD tests).

**Figure 3:**
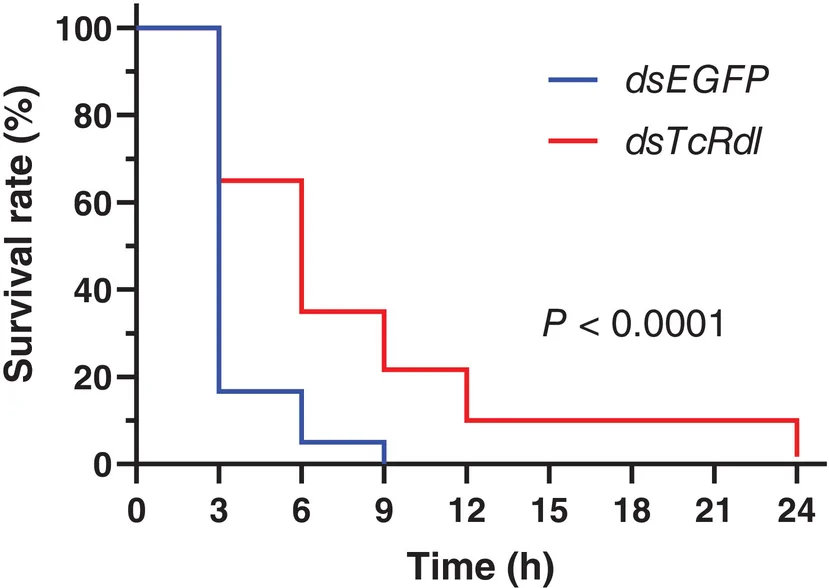
Effects of *TcRdl* knockdown on the fipronil susceptibility in adult *T. castaneum*. The survival rates were compared in topical application method (n = 60) in which 10 nmol μL^−1^ fipronil was applied to ventral surface of abdomen and the number of dead beetles was estimated 3, 6, 9, 12, 24 h post‐application. Differences between survival curves were tested with the log‐rank (Mantel–Cox) test; *P*‐value was indicated on the plot.

In order to identify the target receptor of (‐)‐α‐thujone in *T. castaneum*, we treated the *TcRdl* knockdown using dsRNA microinjection. qRT‐PCR analysis revealed a significant reduction in *TcRdl* transcript levels compared to control counterparts (*F*_3,8_ = 144.1, *P* < 0.001), with the expression decreased by 70% of the untreated counterpart [Fig. 2(A)]. The knockdown beetles showed slight reduction of walking speed but were still sufficiently mobile to move around in petri dish within the test window. The *dsTcRdl* treatment significantly reduced the avoidance behavior toward (‐)‐α‐thujone (*F*_3,16_ = 9.8088, *P* < 0.001) but not toward *l*‐menthol, the monocyclic monoterpenoid compound previously described as an irritant compound^10^ (*F*_3,16_ = 0.0284, *P* = 0.9933) [Fig. 2(B), (C)]. To further investigate the effect of *TcRdl* knockdown, fipronil‐associated mortality was evaluated using a topical application assay. *TcRdl* suppression via RNAi significantly reduced the mortality at each point 12 h post‐application of fipronil than that recorded in *dsEGFP*‐treated beetles, with no significant change detected at 24 h post‐application [Log‐rank (Mantel‐Cox) test; *P* < 0.0001] (Fig. 3).

### 3.3 Effects of *TcTRPL* and *TcTRPM* knockdown on the avoidance behavior toward (‐)‐α‐thujone

**Figure 4:**
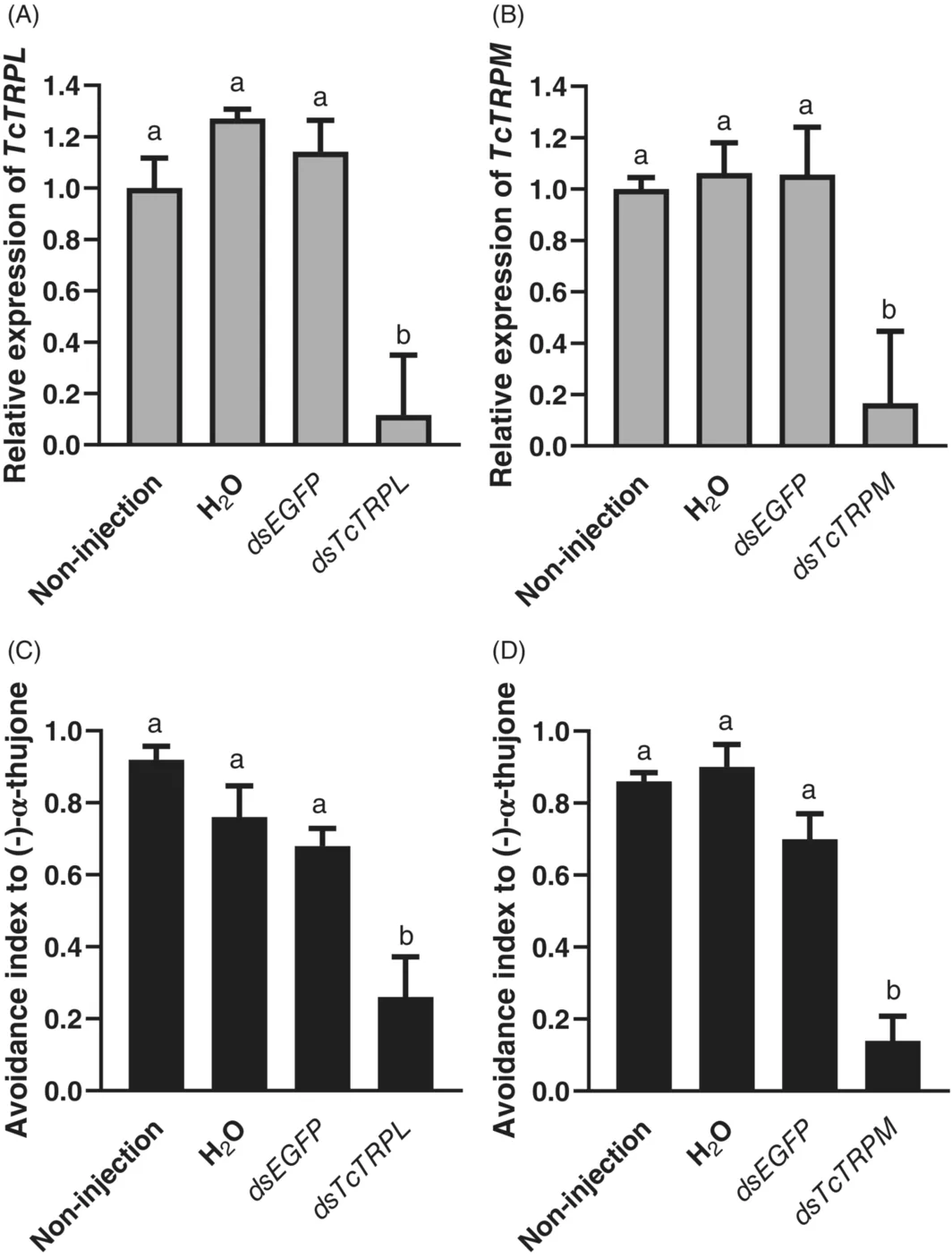
Effects of RNAi‐induced *TcTRPL* and *TcTRPM* knockdown. The knockdown efficiencies of *TcTRPL* (A) and *TcTRPM* (B) were measured via qRT‐PCR. The expression level was expressed as the fold‐change relative to the expression level shown in the noninjected beetles (n = 3). The *TcTRPL* (C) and *TcTRPM* (D) knockdown resulted in a reduction in avoidance behavior toward (‐)‐α‐thujone (n = 5). Data are expressed as mean ± SEM; the same letters above the bars indicate no significant difference at *P* > 0.05 (one‐way ANOVA and Tukey–Kramer HSD tests).

Some cyclic monoterpenoid compounds were considered potential targets of TRP channels.^21^ We investigated the involvement of TcTRPL and TcTRPM channels in the avoidance behavior toward (‐)‐α‐thujone. qRT‐PCR analysis confirmed the suppression of *TcTRPL* and *TcTRPM* expression via RNAi treatment (*F*_3,8_ = 57.336, *P* < 0.001; *TcTRPL*, *F*_3,8_ = 24.382, *P* < 0.001; *TcTRPM*) [Fig. 4(A), (B)], and both groups of beetles exhibited a significant reduction in the avoidance behavior compared with the control (*F*_3,16_ = 13.217, *P* < 0.001; *dsTcTRPL*, *F*_3,16_ = 9.0514, *P* < 0.001; *dsTcTRPM*) [Fig. 4(C), (D)].

### 3.4 Relative expression levels of *TcRdl* and *TcTRPL*

**Figure 5:**
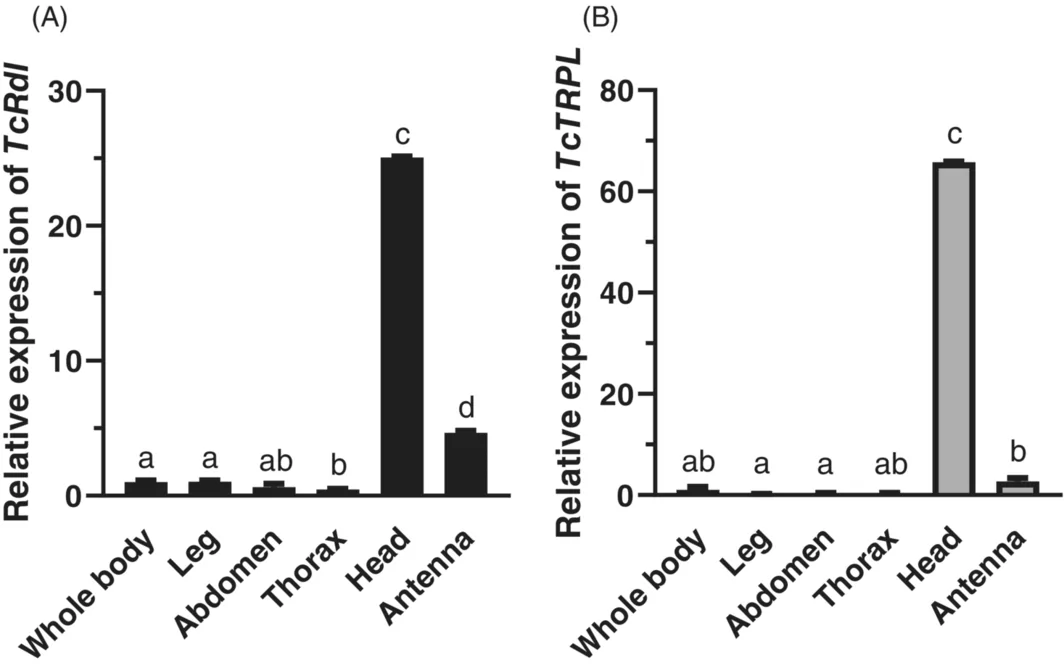
Spatial patterns of (A) *TcRdl* expression indicated dominant expression in head, followed by antenna, and (B) *TcTRPL* expression indicated prominent expression in head. The expression level in each organ of adult *T. castaneum* was revealed as the fold‐change relative to the expression level in the whole body extract (n = 3); data were expressed as mean ± SEM. The same letters above the bars indicate no significant difference at *P* > 0.05 (one‐way ANOVA and Tukey–Kramer HSD tests).

Rdl and TRPL are expressed dominantly in the central nervous system (CNS) and compounds eyes of adult insects, respectivity.^22^, ^23^, ^24^ We compared the spatial expression profile of *TcRdl* and *TcTRPL* transcripts across five regions: leg, abdomen, thorax, head and antenna; the head exhibited high *TcRdl* transcript expression, followed by antenna (*F*_5,12_ = 107.72, *P* < 0.001), and the head also prominently exhibited high *TcTRPL* expression (*F*_5,12_ = 27.94, *P* < 0.001) [Fig. 5(A), (B)].

### 3.5 Involvement of the antenna in avoidance behavior toward (‐)‐α‐thujone

**Figure 6:**
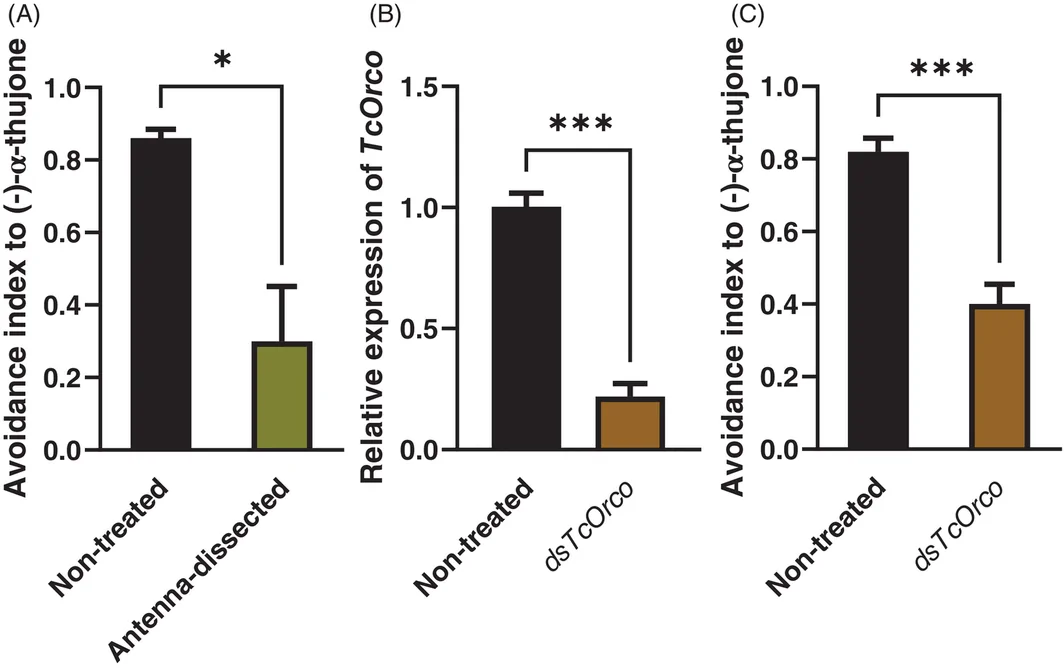
Involvement of antenna in the olfactory perception associated with avoidance behavior toward (‐)‐α‐thujone in *T. castaneum*. (A) Avoidance behavior of antenna‐dissected beetles was compared through the area‐preference test (n = 5). (B) Olfactory receptor co‐receptor (*TcOrco*) knockdown efficiency was confirmed via qRT‐PCR (n = 3), (C) and avoidance behavior of the beetles were compared with nontreated beetles (n = 5). Data are expressed as mean ± SEM; asterisks above the bars indicate significant difference compared with the nontreated beetle: *, *P* < 0.05 (Welch's *t*‐test) (A); ***, *P* < 0.001 (Student's *t*‐test) (B), (C).

In order to clarify the involvement of the antenna in avoidance behavior toward (‐)‐α‐thujone, the comparative area‐preference test using the antenna‐dissected and *dsTcOrco*‐treated beetles along with nontreated beetles was conducted. The antenna‐dissected beetles revealed a significant suppression of avoidance behavior by approximately half of the control counterpart (Welch's *t*‐test, *P* < 0.05) [Fig. 6(A)]. The *TcOrco* knockdowned beetles also exhibited reduced avoidance behavior (Student's *t*‐test, *P* < 0.001) [Fig. 6(B), (C)].

## 4 DISCUSSION

This study demonstrated that (‐)‐α‐thujone acts as an irritant compound for *T. castaneum*. To elucidate the target molecules, we performed area‐preference tests and confirmed the decline in avoidance behavior of beetles toward (‐)‐α‐thujone using RNAi‐based knockdown of three types of candidate target receptors and channels. GABA‐gated chloride channel consisting of the Rdl subunit, widely distributed in the nervous system, is associated with inhibitory‐transmitter receptor throughout insects and related arthropods, including *T. castaneum*.^25^ In a previous study, thymol, a monocyclic phenolic compound derived from *Thymus vulgaris*, potentiated GABA responses on the *Drosophila* Rdl receptor, heterologously expressed in *Xenopus laevis* oocyte, which represented an avenue for pest management.^26^ The present study revealed that *dsTcRdl*‐treated beetles declined avoidance behavior toward (‐)‐α‐thujone, potentially identifying it as a target receptor. Insect Rdl receptor is considered a pesticide target since it was initially discovered in *D. melanogaster*.^27^ In topical application treatment of fipronil, *TcRdl* knocking down beetles exhibited time delay until they resulted in death because fipronil could not bind the target TcRdl. Meanwhile, another target of fipronil was reported in insect (e.g. glutamate‐activated chloride channel^28^); hence, we suggest that the knockdown did not affect the mortality at 24 h post‐application in our experiment.

TRP channels are targets for some plant‐derived components, which contain a cyclic structure, for instance, carvacrol, a structural isomer of thymol, inhibited *Drosophila* TRPL channel stably expressed in S2 cells,^29^ and menthol, monocyclic monoterpenoid, induced TRPM channel‐mediated avoidance behavior in insects,^10^, ^30^ suggesting that this structural property would be important. However, little is known about bicyclic compound as a target of TRP channels. To reveal the structural redundancy of cyclic monoterpenoid as an irritant compound associated with these two TRP channels, (‐)‐α‐thujone was tested using the *dsTcTRPL*‐ and *dsTRPM*‐treated beetles, which exhibited significant reduction in the avoidance behaviors. Insect TRPL channel, belonging to the TRPC subfamily, participates in phototransduction as a light‐activated channel; TRPM channel, a thermo TRP channel, serves as the cold sensor and is involved in magnesium and zinc (Mg^2+^ and Zn^2+^) homeostasis.^31^, ^32^ To the best of our knowledge, this is the first report of TRPL‐mediated avoidance behavior against a plant‐derived compound; intriguingly, both the thermo and non‐thermo TRP channels are targets of (‐)‐α‐thujone.

Our previous reports revealed that *TcTRPM* transcripts were broadly expressed without noticeable organ‐specific expression although *TcOrco* were prominently expressed in antenna.^9^, ^10^ Here, *TcRdl* transcripts were the second most expressed factor in antenna that was followed by head, and *TcTRPL* transcripts were significantly expressed in head, revealing the different expression patterns among them. Both antenna‐dissected and *dsTcOrco*‐treated beetles showed significantly reduced avoidance behavior. Two *Orco* mutant *D. melanogaster* lines exhibiting decreased repellency behavior toward α‐thujone,^17^ together with our data, imply that the antenna‐mediated avoidance behavior is Orco‐dependent, indicating an olfactory‐receptor‐concerned mechanism; however, it may not be the dominant target in *T. castaneum* because *TcOrco* knockdown beetles still exhibit weak avoidance behavior toward (‐)‐α‐thujone. Instead, the dominant expressions of *TcRdl* and *TcTRPL* in head (probably CNS for *TcRdl*) would be another targets, further functional analysis will be desirable.

Overall, the targets of (‐)‐α‐thujone mediating avoidance behavior in *T. castaneum* indicated multimodality. DEET has been most widely used as an insect repellent since its discovery in the 1940s, and considerable efforts have been made to identify DEET receptors to encourage the development of new repellents, in which the multimodal targets: some olfactory receptors and gustatory receptors have been identified in dipteran species.^33^ Notably, different insect species respond to a repellent (e.g. DEET) differently,^6^ and the multimodal targets may be an advantageous factor as repellent. This study is limited to the behavioral data from *T. castaneum*, and further research at the molecular or cellular levels to clarify the interaction between (‐)‐α‐thujone and each receptor can elucidate the underlying mechanisms.

## 5 CONCLUSIONS

We identified TcRdl, TcTRPL, TcTRPM and TcOrco as potential multimodal targets of (‐)‐α‐thujone based on the behavioral approach in *T. castanuem*. The results provide valuable insights into the mechanism of MoA of (‐)‐α‐thujone, which can facilitate the development of new effective strategies for protecting crop plants from the pests.

## CONFLICT OF INTEREST

The authors declare no conflict of interest.

## Supporting information


**Table S1.** Primer sequences used in this study.

## Data Availability

The data that support the findings of this study are available from the corresponding author upon reasonable request.
