# Supplementary material for: Multimodal targets of (‐)‐α‐thujone mediating avoidance behavior of a stored‐products pest, Tribolium castaneum , in response to irritant stimuli
Source: Pest Manag Sci. 2026 Jun 8;82(10):9592–8. doi: 10.1002/ps.70995 (PMC13569170; doi:10.1002/ps.70995)
Supplement: Supplementary file 1 [file PS-82-9592-s001.docx]

Supplementary Table 1. Primer sequences used in this study

| Gene | Accession No. |  | Primer (5' - 3' ) | Size (bp) |
| --- | --- | --- | --- | --- |
| PCR |  |  |  |  |
| *TcRdl* | TC010482 | Forward | ATGGGGCACAGCCGGGTG | 1449 |
|  |  | Reverse | TTACTTATCCTCCTCCAGAAGCACTAGATC |  |
|  |  |  |  |  |
| *TcTRPL* | TC007743 | Forward | ATGGGGGATTCGGTTGATGTTG | 3636 |
|  |  | Reverse | TTAAAGCCAGCCAGTTTTTGGC |  |
|  |  |  |  |  |
| dsRNA | |  |  |  |
| *TcRdl* |  | Forward | TAATACGACTCACTATAGGGCTTGGGCGACGTCAACATATCG | 556 |
|  |  | Reverse | TAATACGACTCACTATAGGGTAGCACCTTAAATTGCGGCAGC |  |
|  |  |  |  |  |
| *TcTRPL* |  | Forward | TAATACGACTCACTATAGGGTCATCTACCAACCCCCTCCAGA | 475 |
|  |  | Reverse | TAATACGACTCACTATAGGGAACGACACATCATCAGAGGGCA |  |
|  |  |  |  |  |
| qRT-PCR |  |  |  |  |
| *TcRdl* |  | Forward | TCGGTTACACCATGCGAGACAT | 137 |
|  |  | Reverse | TGTGGTGAGGCTGATCTCCATG |  |
|  |  |  |  |  |
| *TcTRPL* |  | Forward | AATCATGCCCAACTTGCCCAAG | 88 |
|  |  | Reverse | GCGAACATTTGCCATGTCTCCA |  |

Underline represents the T7 promoter sequence.
